# Correlation of low birth pH with cognitive and language outcomes at two years

**DOI:** 10.3389/fped.2025.1654282

**Published:** 2025-07-31

**Authors:** Khaled Abdelrahman, Anne Groteklaes, Sebiha Demir, Jorge Jiménez Cruz, Till Dresbach, Andreas Mueller, Hemmen Sabir

**Affiliations:** ^1^ Department of Neonatology and Pediatric Intensive Care Medicine, University Hospital Bonn, Bonn, Germany; ^2^ Department of Obstetrics and Prenatal Medicine, University Hospital Bonn, Bonn, Germany

**Keywords:** perinatal acidosis, outcome, encephalopathy, PARCA-R, neurodevelopment

## Abstract

**Background:**

Mild hypoxic-ischemic encephalopathy (HIE) often goes undected due to the narrow therapeutic window, subtle or absent clinical signs, and the lack of standardized diagnostic criteria. A broader umbilical cord blood pH threshold may help identifying at-risk neonates. Long-term developmental outcomes of term-born infants with isolated acidosis are lacking.

**Objective:**

To examine the long-term non-verbal cognitive and language development at two years of term-born infants with an umbilical cord blood pH between 7.0 and 7.15 but no apparent clinical signs of mild HIE.

**Study design:**

Retrospective observational study of 101 term-born infants. Developmental outcome was assessed using the Parent Report of Children's Abilities—Revised (PARCA-R) questionnaire for infants aged between 23 months and 27 months. Standardized PARCA-R scores are interpreted as follows: ≥85 = normal development, 70–84 = mild developmental delay, <70 = moderate developmental delay.

**Results:**

PARCA-R Scores <85 in either non-verbal cognitive or language delay occurred in 39% of the cohort: 32% of them in non-verbal cognitive and 16% in language domain. No significant differences in developmental outcomes between the pH subgroups were found. Our outcomes were highly consistent with reported outcomes of infants diagnosed with mild HIE.

**Conclusion:**

Term-born infants with isolated perinatal acidosis may be at increased risk of developmental impairments at two years, despite being considered clinically healthy at birth.

## Introduction

Perinatal asphyxia, an acute oxygen deficiency affecting the vital organs of newborns before, during, or shortly after birth, is one of the leading causes of neonatal mortality (1). When moderate to severe perinatal asphyxia occurs, it can result in hypoxic-ischemic encephalopathy (HIE) (2). HIE is characterized not only by pure hypoxia—a mismatch between oxygen uptake and delivery—but also by ischemia, where insufficient tissue perfusion threatens tissue necrosis (2–4).

Predicting which cases of neonatal asphyxia will progress to HIE is challenging. HIE is commonly identified using inclusion criteria such as perinatal acidosis (umbilical artery pH < 7.0), an APGAR score ≤ 5 at 10 min, and the necessity for resuscitation lasting ≥10 min. Clinical (Sarnat score) and electrophysiological (aEEG) criteria allow for the recognition of HIE within the therapeutic window of 6 h (4–6).

During that critical therapeutic window—the time span before the onset of irreversible cell damage—HIE must be promptly diagnosed and interventions to prevent or minimize cell death should be initiated. While newborns with moderate to severe HIE are well-characterized and routinely treated with therapeutic hypothermia, the recognition and impact of mild HIE remains in a diagnostic gray zone. Infants with mild HIE often do not meet the neurological criteria for therapeutic hypothermia and are generally perceived to have favorable outcomes. Consequently, they are excluded from structured follow-up protocols (5–7).

This perception, however, is increasingly being challenged by new data showing that even infants with clinically mild symptoms may later develop significant neurodevelopmental impairments. Studies such as those by Chalak et al. and Murray et al. highlight that children diagnosed with mild HIE exhibit cognitive and behavioral deficits at two and five years of age comparable to or only slightly better than those of moderate HIE. In addition, magnetic resonance imaging (MRI) abnormalities have been reported in a substantial number of infants with mild HIE, supporting the concern that neurological injury may already be present despite mild clinical findings (7, 8). Thus, clinical diagnostic tools such as the Sarnat staging system may often not be sensitive enough to detect early, subtle signs of brain damage consistent with mild HIE (5–8).

Due to the narrow therapeutic window for diagnosing and treating HIE, signs of brain damage may only be recognized after the opportunity for effective treatment has passed. For this reason, there is growing interest in the use of additional tools to help detect at-risk newborns earlier (5, 6). One such tool is the measurement of pH in umbilical cord blood, which is a metabolic marker for perinatal distress.

Umbilical cord blood pH is a widely available and objective marker of perinatal asphyxia. A pH below 7.0 is generally considered as an indicator of significant acidosis and fetal distress and is commonly used in clinical practice to guide treatment decisions. However, pH values between 7.0 and 7.15, while still outside the target normal values, are often below the threshold for concern and frequently overlooked (9). This pH range is of particular interest to us here, as it does not yet require therapeutic hypothermia or additional follow-up, but still represents perinatal acidosis by definition. Large-scale epidemiologic studies further demonstrated that even higher pH threshholds, reaching up to 7.24, are associated with an increased risk of neonatal complications, including respiratory distress, seizures, and later cognitive impairment (10–12).

Nevertheless, there are few studies that specifically examined the long-term developmental outcomes of infants whose only indicator of perinatal distress is an umbilical cord blood pH within this range. These infants are typically not diagnosed with mild HIE, are not treated with therapeutic hypothermia, and therefore do not usually participate in follow-up programs. Thus, they may represent a clinically silent but potentially vulnerable population. Our study aims to address this gap by evaluating the cognitive and language development of term-born infants with an umbilical cord blood pH between 7.0 and 7.15 using the Parent Report of Children's Abilities—Revised (PARCA-R) questionnaire for infants between 23 and 27 months of age.

We hypothesize, despite the absence of clinical neurological signs at birth, that this metabolically defined subgroup is nevertheless at increased risk for subtle developmental deficits. By focusing on this under-investigated group, our study aims to challenge existing assumptions about neonatal risk and contribute to a better understanding of the perinatal factors that influence long-term developmental outcomes.

## Methods & material

### Study design and population

We designed and conducted a retrospective observational study using data from infants born at Bonn University Hospital. The study was approved by the local ethics committe (nr. 472/22-E). Of 172 eligible infants, 101 term-born infants (gestational age ≥ 37 weeks) born between June 2022 and February 2023 participated in our study, all of whom had arterial umbilical cord blood pH values between 7.0 and 7.15. The arterial umbilical cord pH value was used as the sole criterion in the present study, infants with congenital malformations or severe intrauterine growth restriction were excluded (Table 1).

**Table 1:** Perinatal characteristics of the cohort for children aged between 23 months and 27 months (*n* = 101). Data are presented as median (IQR).

Parameter	Perinatal characteristics
Gestational age (weeks)	39.00 (38.43–40.00)
Birth weight (g)	3,490 (3,214–3,742)
Male	53
Apgar scores (min)	
1	8 (7–9)
5	9 (9–10)
10	10 (10–10)
Umblicial cord blood—pH	7.12 (7.09–7.13)
Umbilical cord blood—base deficit (mmol/L)	−10.7 (−12.5 to −9.1)

None of the infants included in this study showed clinical signs of HIE at birth, and none received therapeutic hypothermia. None of the them required postnatal hospitalization beyond routine postnatal care, nor were they admitted due to complications or symptoms suggesting encephalopathy. In addition, they were not admitted or treated in hospital until the developmental assessment. Due to the absence of any clinical indication of HIE, standard neurological assessments such as Sarnat scoring, amplitude-integrated EEG (aEEG), or brain imaging were not performed. This allowed us to investigate a group of infants with metabolic evidence of perinatal acidosis but no clinical diagnosis of HIE.

### Developmental assessment with PARCA-R

Developmental outcomes were assessed using the Parent Report of Children's Abilities—Revised (PARCA-R). The PARCA-R is a standardized questionnaire designed for use for children aged between 23 months 16 days and 27 months 15 days to evaluate cognitive and language development based on parental observations. It has been validated against established developmental tests such as the Bayley Scales of Infant Development (BSID-II and Bayley-III) and has shown good reliability and concurrent validity, particularly among infants born preterm or infants at increased risk for neurodevelopmental disorders (13, 14).

The designed questionnaire includes two main sections: the first assessing non-verbal cognitive development (scored 0–34), and the other is to follow up language development (scored 0–124). The raw scores obtained from each section were converted into standardized scores using age- and sex-specific reference tables developed from a large UK cohort. These standardized scores have a mean of 100 and a standard deviation of 15 (15).

Standardized PARCA-R scores are interpreted as follows:

• Scores ≥85: Normal development
• Scores between 70 and 84: Mild developmental delay
• Scores <70: Moderate developmental delay
• Scores <54: Severe developmental delay

These thresholds are consistent with those used in prior clinical and epidemiological research (13–15).

### Outcome definition

The primary outcome is the above-mentioned developmental status at two years of age, as measured by the standardized cognitive and language scores derived from the PARCA-R questionnaire. Parents of the participating children were contacted and the PARCA-R questionnaire was sent to them. In the following week, the entire PARCA-R questionnaire was completed in a structured interview conducted by the investigator. During the interview, parents answered the questionnaire components in real time, and the investigator recorded the responses directly.

Following each interview, raw scores for language development and cognitive development were calculated according to the official PARCA-R scoring instructions (15). These raw scores were then converted into the standardized scores. The German PARCA-R questionaire was used and all interviews were conducted in German.

The resulting standardized scores were categorized into developmental outcome groups according to predefined thresholds: normal development, mild delay, moderate delay and severe delay.

### Statistics

Statistical analysis was performed using GraphPad Prism (Version 10.4.1). We first tested for normal distribution of data using Shapiro–Wilk-test. All data were normally distributed. We then tested correlation between umbilical cord blood pH and PARCA-R scores using Pearson's correlation. We further used logistic regression to analyze whether cord blood pH predicted PARCA-R scores and if there was any influence of sex or birth weight. The significance level was set to a *p*-value of <0.05.

## Results

A total of 101 term-born infants with umbilical cord blood pH between 7.0 and 7.15 were included in the final analysis. The cohort was further divided into four subgroups to investigate potential trends at different pH thresholds:

• First cohort: pH < 7.1 (*n* = 32)
• Second cohort: pH ≥ 7.1 (*n* = 69)
• Third cohort: pH ≤ 7.1 (*n* = 41)
• Fourth cohort: pH > 7.1 (*n* = 60)

The whole monitored group is referred to as full cohort (*n* = 101) with pH ≥ 7.0 and <7.15.

Each infant was evaluated at approximately two years (23–27 months) using the PARCA-R questionnaire. Standardized scores for both cognitive and language development were calculated and categorized as follows: normal (≥85), mild delay (70–84), moderate delay (<70) and severe delay (<54).

### Cognitive scores

In the full cohort (*n* = 101), 27% of the infants demonstrated mild cognitive developmental delay (PARCA-R scores 70–84), while 5% scored below 70, indicating moderate developmental delay.

A breakdown of cognitive outcomes by pH subgroup is presented in Table 2. Across all four subgroups, the distribution of cognitive scores remained consistent, with no trend or shift in developmental status as a function of pH. For example, in the first cohort (pH < 7.1), 31% scored 70–84 and none scored below 70, while in the second cohort (pH ≥ 7.1), 25% scored 70%–84% and 7% scored below 70. Similar ratios were observed in the third and fourth cohorts.

**Table 2:** Developmental outcomes using the parent report of children's abilities-revised (PARCA-R).

Standard score	Cognitive score	Language score
Full cohort results (*n* = 101) pH ≥ 7.0		
≥85	69 (68%)	85 (84%)
70–84	27 (27%)	15 (15%)
<70	5 (5%)	1 (1%)
First cohort results (*n* = 32) pH < 7.1		
≥85	22 (69%)	26 (81%)
70–84	10 (31%)	6 (19%)
<70	–	–
Second cohort results (*n* = 69) pH ≥ 7.1		
≥85	47 (68%)	59 (86%)
70–84	17 (25%)	9 (13%)
<70	5 (7%)	1 (1%)
Third cohort results (*n* = 41) pH ≤ 7.1		
≥85	28 (68%)	33 (80%)
70–84	12 (30%)	8 (20%)
<70	1 (2%)	–
Fourth cohort results (*n* = 60) pH > 7.1		
≥85	41 (68%)	52 (87%)
70–84	15 (25%)	7 (12%)
<70	4 (7%)	1 (1%)

### Language scores

Language development outcomes followed a similar pattern. In the full cohort, a total of 16% had mild-to-moderate developmental delays.

Again, subgroup analysis revealed no statistically significant differences in language outcomes between infants with a pH above or below 7.1. Each subgroup had proportions of normal and delayed language scores that were very similar to those of the overall population. For example, in the third cohort (pH ≤ 7.1), 80% of infants scored ≥ 85, while in the fourth cohort (pH > 7.1), the figure was 87%.

Notably, none of the children in the study scored below 54 on either cognitive or language subscales, and therefore no infant was categorized as having severe developmental delay. Based on this, the corresponding score category was excluded from Table 2.

### Combined cognitive and language delay

Looking at the combined results (i.e., children with cognitive or language scores <85), 39% of the full cohort showed some form of developmental delay. Table 3 summarizes these results, with no significant differences observed between genders: 38% of male children and 40% of female children were below the developmental threshold in at least one domain.

**Table 3:** Proportion of children with developmental delay, overall and by sex.

Proportion of children with developmental delay, overall and divided by sex	*n*	Non-verbal cognitive delay	Language delay	Non-verbal cognitive or language delay
		*n* (%)	*n* (%)	*n* (%)
Standard score < 85				
Total	101	32 (32%)	16 (16%)	39 (39%)
Males	53	15 (28%)	10 (19%)	20 (38%)
Females	48	17 (35%)	6 (16%)	19 (40%)

None of the analysed parameters correlated significantly with individual umbilical cord pH and umbilical cord pH was not able do predict PARCA-R outcomes (Table 4).

**Table 4:** Pearson correlation.

Correlation between umbilical cord blood pH and PARCA-R scores	pH vs. cognition	pH vs. language
*r*	−0.02470	−0.06972
95% confidence interval	−0.2191 to 0,1716	−0.2616 to 0.1275
*R* squared	0.0006099	0.004861
*P* value		
*P* (two-tailed)	0.8063	0.4884
*P* value summary	ns	ns
Significant? (alpha = 0.05)	No	No

## Discussion

This study presents novel results suggesting that infants born with an umbilical cord blood pH between 7.0 and 7.15, despite being considered clinically healthy at birth, may be at increased risk for developmental delay at 24 months of age. The proportion of infants in our cohort experiencing developmental delays is remarkably similar to the secondary outcomes reported in the PRIME study, despite key differences in the definition of the cohort (7). While the PRIME study included infants with clinical signs of mild HIE confirmed within the first six hours of life using neurological (Sarnat scoring system) and electrophysiological (aEEG) criteria, our cohort was selected solely on the basis of umbilical cord pH values between 7.0 and 7.15. Notably, 32% of the children in our study had nonverbal cognitive scores below 85 (mild to moderate developmental delay) and 39% were either cognitively or language delayed, which is highly consistent with the secondary outcomes observed in the PRIME cohort. None of the infants in our monitored group met criteria for therapeutic hypothermia, showed signs of mild HIE, or received structured follow-up. Yet our use of the validated German version of the PARCA-R questionnaire yielded cognitive and language scores very similar to those obtained in infants with mild HIE. This similarity suggests that umbilical cord pH alone may be a strong early marker of neurodevelopmental risk, even in the absence of clinical symptoms immediately after birth.

According to the validated PARCA-R norms, approximately 15% of children in the general population are expected to score below 85 based on the standard Gaussian distribution (15). In our cohort, however, 32% scored below this threshold in non-verbal cognition and 39% in either cognitive or language domains, indicating more than a doubling of the expected risk for developmental delay. Additionally, our results highlight a consistent high risk for developmental impairment across the pH spectrum from 7.0 to 7.15 that is independent of small differences in metabolic acid-base status at birth.

Umbilical cord blood pH values above 7.0 have so far been considered clinically harmless unless they were accompanied by low Apgar scores or manifest neurological symptoms. However, recent large cohort studies have challenged this view and showed that even mild perinatal acidosis (pH thresholds reaching 7.24) is associated with an increased rate of neonatal complications such as respiratory problems, seizures and long-term developmental problems (10–12). These findings, combined with our current data, suggest that the threshold of pH < 7.0 as a primary indicator of clinically relevant acidosis may be too conservative. Our results support these concerns, as we found no significant differences in developmental outcomes between the subgroups with a pH between 7.00 and 7.15. This suggests a relatively consistent developmental risk in this pH range.

Our findings on infants with mild perinatal acidosis raise concerns about silent long-term risks, similar to neurodevelopmental impairments observed in other clinically manifested mild HIEs (7). The idea these infants represent a silent risk group is supported by studies such as that done by Murray et al., who reported that children with mild HIE can have cognitive deficits almost equivalent to those of children with moderate HIE up to the age of five years (8). As mild cases often lack clear clinical signs, it appears that they are unlikely to be detected using common diagnostic tools such as the Sarnat staging system. In this context, metabolic markers such as umbilical cord blood pH provide an early, objective and measurable indicator of fetal distress. The similarity between the results of our cohort and those of patients with mild HIE emphasizes the value of umbilical cord blood pH in identifying infants who may benefit from closer monitoring.

A major obstacle to the timely treatment of HIE is the narrow but critical time window of 6 h in which HIE must be diagnosed. Once diagnosed in time, interventions such as therapeutic hypothermia must be directly initiated to prevent or minimize cell death in order to have an effective treatment. In this narrow time window, the clinical manifestations of mild HIE may be too subtle to be recognized (5). Such delay in the diagnosis may result in neonates who could benefit from neuroprotective strategies not being adequately recognized and treated. The inclusion of a broader definition of the umbilical cord blood pH threshhold in standard neonatal screening protocols could help to identify infants at risk earlier enough and enable follow-up and support before more serious impairments occur (9). This could help to overcome the oversimplified binary distinction between HIE and non-HIE and eventually recognize the HIE as a broader spectrum of risks and outcomes.

Our results add to the study done by Vesoulis et al. who also examined the utility of a broader definition of umbilical cord blood pH threshold as a screening criterion for neonatal encephalopathy. Their results showed that lowering the pH screening threshold from <7.00 to ≤7.10 significantly increased the sensitivity of identifying infants who developed moderate to severe HIE. More important, a significant proportion of these infants (more than one-third) were only detected by the newly defined pH criterion without meeting traditional inclusion criteria such as Apgar score ≤5 at 10 min or prolonged resuscitation. This suggests that relying solely on traditional clinical indicators may result in under-detection of at-risk neonates (9). Our study extends these findings by demonstrating that term-born infants with umbilical cord blood pH values between 7.00 and 7.15, who similarly appear clinically healthy at birth, may nonetheless experience developmental delays at two years of age.

The developmental risks in this group are not limited to delays in cognitive and language functioning. Previous studies have reported increased rates of behavioral and executive function problems, including a higher risk of ADHD, learning difficulties, memory problems, dyslexia, social-emotional challenges and a lower intelligence quotient (IQ) compared to healthy peers (5, 8, 9, 16–20). These issues are often more noticeable in the early school years, highlighting the need for continued developmental monitoring beyond infancy (5, 8). It is therefore likely that our results still underestimate the true developmental risk associated with isolated perinatal acidosis. Literature on mild HIE suggests that many neurocognitive and behavioral impairments only become apparent in later childhood. Thus, even the detection of delays at two years of age points to a clinically relevant risk that may intensify over time.

Although the reference data were derived from the UK, a German version of the PARCA-R has been validated and showed well correlation with Bayley Scale results (14). Picotti et al. (2020) validated the German-language version of the PARCA-R in a large sample of very preterm infants, demonstrating that the standardized scores derived from the UK reference population correlate well with developmental outcomes assessed using the Bayley Scales. This supports the PARCA-R's cross-cultural validity and clinical utility in the German context. The PARCA-R, being a norm-referenced instrument standardized on a large population-based UK cohort, inherently reflects background factors such as socioeconomic status and parental education within its normative data, thereby addressing potential confounding variables on developmental outcomes (13–15).

Several limitations of our study should be acknowledged. First, although the PARCA-R is a validated and widely used instrument, it is still based on parent reports that may be influenced by subjective factors. Second, our study evaluated outcomes at a single time point (children aged between 23 months 16 days and 27 months 15 days), which may not capture cognitive or behavioral challenges that develop later. Long-term follow-up into school age would provide a more complete picture. Third, while the PARCA-R is pragmatic and particularly appropriate for the aims of this study, it is not equivalent to formal clinical assessments such as the Bayley scales, which remain the gold standard for identifying subtle developmental delays with high precision.

## Conclusion

This study suggests that term-born infants with umbilical cord blood pH values between 7.0 and 7.15, despite being considered clinically healthy at birth, may have an increased risk of developmental impairment by two years of age. These findings point to a potential gap in current neonatal care and suggest that the risk of long-term developmental impairment may be underestimated by relying solely on the current HIE definition. Future prospective studies should focus on monitoring children born with isolated perinatal acidosis beyond infancy to clarify their long-term developmental risk and to better differentiate and define potential developmental impairment.

## Data Availability

The original contributions presented in the study are included in the article/Supplementary Material, further inquiries can be directed to the corresponding author.
